# From Habit-Forming to Habit-Breaking Availability: Experiences on Electronic Gambling Machine Closures During COVID-19

**DOI:** 10.3389/fpsyg.2021.788586

**Published:** 2022-01-20

**Authors:** Virve Marionneau, Johanna Järvinen-Tassopoulos

**Affiliations:** ^1^Faculty of Social Sciences, Centre for Research on Addictions, Control, and Governance, University of Helsinki, Helsinki, Finland; ^2^Finnish Institute for Health and Welfare, Helsinki, Finland

**Keywords:** gambling, EGMs, COVID-19, habit, Finland

## Abstract

Electronic gambling machines (EGMs) are among the most harmful forms of gambling. The structural characteristics of EGMs prolong and reinforce gambling similarly to other habit-forming technologies. In Finland, the wide availability of EGMs in non-casino locations is likely to further reinforce the habit-creating nature of gambling offer by incorporating EGMs into everyday practices. The COVID-19 pandemic changed the landscape of gambling in Finland. The most visible change was the closure of land-based EGMs in non-casino environments, arcades, and the casino in March 2020. Since then, the status of EGMs has varied depending on the pandemic situation. The current qualitative study focuses on how Finnish past-year gamblers experience prolonged EGM closures and occasional re-openings 1 year into the pandemic. The data consist of responses to an online questionnaire eliciting experiences (*N* = 187) as well as interviews (*N* = 27, conducted in groups or alone). To aid our analysis, we employ the sociological pragmatist theory of the concept of “habit.” The analysis focuses on gambler experiences on EGM shutdowns and re-openings, and views on whether closures have contributed to abstaining from gambling or to shifting to other gambling products. Policy implications of the results are discussed.

## Introduction

The gambling landscape changed dramatically during the COVID-19 pandemic in Finland, as elsewhere. The Finnish gambling field is strongly characterised by non-casino EGMs that are placed in business premises such as supermarkets and petrol stations, or in dedicated gambling arcades located in shopping centres. Regulated gambling is provided by monopoly-holder Veikkaus. Before the COVID-19 pandemic, EGMs constituted a significant share of the gross gambling revenue of Veikkaus: Over half of the gross gambling revenue of the monopoly holder were derived from so-called excitement games consisting mainly of EGMs (Marionneau and Lähteenmaa, 2020). Although Veikkaus is in the process of reducing its EGM network, Finland still has a high per capita count of EGMs (Heiskanen et al., 2020). EGM gambling is also prevalent in Finnish population studies. The population study of 2019 shows that 31 percent of Finns had gambled on non-casino EGMs during the last 12 months (Salonen et al., 2020). The share is notably higher than for example in the neighbouring countries Sweden or Norway (two and five percent, respectively, cf. Heiskanen et al., 2020).

EGMs are one of the most harmful forms of gambling. In Finland, as well as in other countries, EGM gambling features predominantly in help-seeking statistics (Livingstone et al., 2019; Heiskanen et al., 2020). Population studies show that participation in EGM gambling is particularly prevalent amongst those who play problematically or who experience gambling harms (Vasiliadis et al., 2013; Sulkunen et al., 2019). The structural characteristics of EGMs are designed to prolong and reinforce gambling. Like other habit-forming technologies, they are designed to maximise the consumer’s time on device even at the risk of harms and addictive behaviours (see Schüll, 2012; Langvardt, 2019). EGMs have been argued to form repetitive habits in consumers (Griffiths and Wood, 2001) owing to characteristics that prolong gambling sessions such as near misses, bonus features, and bonus ladders, but also ergonomic design (Schüll, 2012). Habit-forming technologies—or even persuasive technologies (cf. Langvardt, 2019)—such as EGMs, are harmful when used, but possibly even more harmful when constantly and widely available. EGMs placed in convenience locations may contribute to habit-formation. When EGMs are readily available in locations such as supermarkets, petrol stations, or other everyday spaces, gamblers will not only be tempted to prolong the duration of play sessions, but also to play more frequently and to incorporate EGM play into everyday practices (Pöysti, 2014; Livingstone et al., 2019; Järvinen-Tassopoulos et al., 2021).

During the first wave of COVID-19 (March 2020 to July 2020), EGMs placed in Finnish non-casino locations, gambling arcades, and Casino Helsinki were shut down. EGMs were reopened in July 2020 but were closed again during the autumn in regions classified as being in an acceleration or community transmission phase of COVID-19, including the Helsinki region. The subsequent waves of COVID-19 have been characterised by closures and partial re-openings of the EGM network depending on the region. These changes are likely to have impacted how gamblers have experienced the availability of EGM provision as well as the role of EGMs in the Finnish gambling landscape.

International studies on the impacts of the COVID-19 pandemic on gambling have indicated that the precautions taken to limit the spread of the coronavirus, including EGM and gambling venue closures, have lessened the overall burden of harms caused by gambling. Restrictions on access have reduced total consumption and experienced gambling harms at a population level (e.g., Brodeur et al., 2021; Hodgins and Stevens, 2021). Evidence from Australia also shows that some reductions in overall gambling involvement have been maintained after restrictions were lifted (Black et al., 2021). However, reductions in total consumption and harms appear to vary depending on the scope of pandemic-related restriction. A representative Italian population study (*N* = 6,003) shows that the prevalence of land-based gambling reduced by 76 percent during the first strict lockdown in Italy in spring 2020. In the same study, the prevalence of online gambling also declined by 20% during the same period. In Sweden, where COVID-19 restrictions have been more lenient with lesser impacts on land-based gambling offer, only 7% or respondents in a panel study (*N* = 2,000) reported less gambling during the first wave of COVID-19 (Håkansson, 2020). During the second wave in autumn 2020, only 4% of Swedish respondents in a similar panel study (*N* = 2,029) reported reduced gambling during the pandemic while 6% reported increased gambling (Håkansson and Widinghoff, 2021).

Despite overall reductions in total consumption in most jurisdictions, some sub-groups of gamblers have also increased their gambling. Increased gambling during lockdowns has been connected particularly to problem gambling severity, young age, male gender, other substance use, and compromised mental health (Hodgins and Stevens, 2021; Lugo et al., 2021). Reductions in land-based gambling consumption have, for some, translated into increases in online gambling (Håkansson, 2020; Lindner et al., 2020; Gainsbury et al., 2021; Georgiadou et al., 2021; Hodgins and Stevens, 2021). In a Canadian population study, overall levels of problematic gambling decreased during lockdown, but online gambling was a prediction for gambling problems during the lockdown period. Seventeen percent of respondents (*N* = 3,449) reported shifting to online gambling (Shaw et al., 2021). Similarly, in a US-based online panel of past 3-month gamblers (*N* = 424), 15% reported migrating to online gambling. Those who had migrated also had higher levels of problematic gambling behaviour (Xuereb et al., 2021).

Finnish studies investigating gambler experiences during the early months of the pandemic show similar results. During spring 2020, gamblers expressed relief over EGM closures (Järvinen-Tassopoulos et al., 2020; Marionneau and Järvinen-Tassopoulos, 2021). Gambling-related harms reduced, and land-based EGM gambling was not substituted by online gambling at a population level (Taloustutkimus, 2020; Marionneau and Järvinen-Tassopoulos, 2021). The annual reports of the Finnish gambling monopoly Veikkaus (2020, 2021) show that while sales of land-based gambling were impacted by the closure of land-based EGMs, the total revenue from the online channel did not rise to a similar degree (land-based sales change from 1,153 M Euros in 2019 to 714 M Euros in 2020; online sales change from 537 M Euros in 2019 to 546 M Euros in 2020). Furthermore, estimations of intelligence company H2 gambling capital (cited in Veikkaus, 2020) show that the share of offshore online gambling has not risen significantly between 2019 and 2020.

What is not yet known is how Finnish gamblers have experienced the prolonged EGM closures and partial re-openings during the extending COVID-19 pandemic time. The current study focuses on how the change from habit-creating and constant availability of EGMs to “habit-breaking” closures is experienced amongst gamblers 1 year into the pandemic.

To study how gamblers experienced changes in the habitual provision of EGM gambling, the current study employs the concept of “habit” as understood in pragmatist social theory. The sociological understanding of habit differs from the psychological understanding of habit as a non-cognitive or individual behavioural structure (cf. Triandis, 1977). The pragmatist habit-based concept of action is rather a social theory of action that helps us understand the predispositions for acting in specific ways. Within a society, individuals tend to follow familiar course of action because alternatives are not actively considered when this is not necessary (Joas, 1996). Habits are learned by being exposed to the practices of others in similar situations (Törrönen et al., 2019). Habits could then be described as dispositions to specific ways of action rather than repetition of actual acts (Kilpinen, 2012). However, habits are also creative, and individuals can adapt them to changing conditions or new contexts (cf. Joas, 1996; Dalton, 2004; Kilpinen, 2012). This creates social processes where changing conditions also change habitual patterns or predispositions of action, and create new adaptions (Kilpinen, 2012). Sulkunen (2009) has called this the “habit cycle” that is only broken when something unexpected happens. Applied to gambling consumption, these theoretical premises suggest that gambling provision may also create such dispositions to act in specific ways. Individual gamblers learn specific dispositions in their social environment, but these habitual practices can change when situations or conditions change (cf. Törrönen et al., 2019 in alcohol studies). Limited availability, as during COVID-19, may therefore be a crucial way to reduce both exposure to gambling and the experience of individuals.

In the following, we will introduce the data, methods, and theoretical premises of the study. The results are presented by focusing on four themes: experiences of prolonged EGM closures, experiences of partial re-openings of EGMs, maintaining non-gambling, and shifts to online gambling.

## Data and Methods

### Sampling and Sampling Procedures

The qualitative data for the current analysis consist of two separate sources: responses to an online questionnaire (*N* = 187) as well as interviews (*N* = 27, conducted in groups or alone). Both sets of data were collected during spring 2021 when non-casino EGMs were again closed in most of the country. The two data sets were collected separately, resulting in somewhat differing sampling. Participants were not screened for problem gambling with relevant instruments (e.g., SOGS, PGSI) because the aim was not to conduct a clinical study but to elicit experiences. For this purpose, self-identified experience of gambling (including problems) was deemed more appropriate.

The online questionnaire was organised in collaboration by the University of Helsinki and the Finnish Association for Substance Abuse Prevention. It was open between February 16th and March 21st, 2021. The questionnaire was promoted widely online, including social media, newsletters, gambling help organisations, and websites of the organising institutions. The questionnaire was addressed to over 15-year-old past year gamblers and concerned significant others (CSOs) of gamblers. Only the responses of gamblers (*N* = 187) are included in the current analysis. As the research focuses mainly on experiences of EGMs, the analysis focused particularly on those who had gambled on EGMs in the past year (*N* = 31). The answers of non-EGM gamblers were considered insofar as they related to EGM gambling before the pandemic (more than 1 year ago), to general observations related to EGM gambling, EGM availability, or the Finnish EGM policy that were detected in the qualitative analysis. Sample characteristics of the online questionnaire respondents are described in Table 1.

**TABLE 1:** Sample characteristics of online questionnaire respondents (*N* = 187).

Sample characteristics	N	Percentage (%)
Gender		
Female	94	50.3
Male	92	49.2
Prefer not to say	1	0.5
Language		
Finnish	184	98.4
Swedish	3	1.6
Age		
15–17	1	0.5
18–24	15	8.0
25–34	21	11.2
35–49	69	36.9
50–64	55	29.4
65–74	21	11.2
Over 74	5	2.7
Education level		
Secondary school	14	7.5
High school or equivalent	86	46.0
University diploma	87	46.5
Region of residence		
Helsinki metropolitan area	14	7.5
Other southern Finland	56	29.9
Western Finland	52	27.8
Eastern Finland	47	25.1
Northern Finland and Lapland	18	9.6

The questionnaire included questions on how the prolonged pandemic situation and related restrictions had impacted respondents’ gambling consumption and habits, experiences of particularly EGM and online gambling during the pandemic, views on treatment and prevention of gambling problems during the pandemic, and how respondents related to EGM closures. Basic background information on the respondents was also collected. The questionnaire was available in a similar form in Finnish and in Swedish (the two official languages of Finland). Questions included both multiple choice and open-ended questions. As the number of participants in the online questionnaire was small, the focus in the analysis was on the answers to open-ended questions. Quantitative data of the material is only given to illustrate more general trends in the data.

Participants in the qualitative interviews were recruited among respondents in the 2020 or 2021 online questionnaires who left their contact information, from gambling-specific websites, and with the assistance of help and treatment organisations. The criterion for participation was to identify as active gamblers or as experiencing problems with their gambling Interviews were organised in March 2021. Recruited participants included gamblers and concerned significant others, but only interviews with gamblers are considered in the current analysis (*N* = 27). The potential interviewees were recruited with a letter explaining the purpose of our study and the process of data collection. Later, the interviewees received another letter informing them of data protection, the anonymisation of the data and the use of the data for scientific purposes. Before starting the remote group or individual interview, the interviewer remined the interviewees of the study and asked again if they were willing to participate in the study.

Similarly to the online questionnaire data, interviews focused on how participants had experienced gambling during the prolonged COVID-19 situation, how they related to EGM availability during the pandemic, how possible changes in gambling had impacted their lives during COVID-19, and what kind of help and treatment services participants thought would be needed for gamblers or CSOs during or after COVID-19. As participants were from various Finnish regions, the interviews also focused on geographical differences in COVID-19 related restrictions.

Seven interviews were organised in a group setting of two to five participants. Three interviewees preferred to be interviewed alone. Group interviews lasted approximately 50–75 min while individual interviews lasted approximately 30 min. Of the 27 participants in the final sample, five identified as recreational gamblers and 22 identified as problem gamblers or as recovered/recovering problem gamblers. 14 males and 13 females participated. Most of the interviewees were between 30 and 65 years old. Characteristics of the interviewees are described in Table 2. Interviewees can be distinguished by their participation in the interview (G = group, P = participant, I = individual), their self-identified gambler type (recreational/problem gambler), gender, age group, and residential area. We use wide age groupings (18–29, 30–65 and 65 + years old) to guarantee the anonymity of the interviewees. For the same reason, the regions of residence have been classified into wider categories. As data collection for the interviews was conducted separately from the questionnaires, these descriptive demographic characteristics are also somewhat different across the samples.

**TABLE 2:** Characteristics and identifiers of interviewees.

Interviewee	Gambler type (self-identified)	Gender	Age group	Region of residence
G1-P1	Recreational	Male	30–65	Southern Finland
G1-P2	Recreational	Male	30–65	Northern Finland
G1-P3	Recreational	Male	30–65	Helsinki metropolitan area
G2-P1	Problem	Male	30–65	Southern Finland
G2-P2	Problem	Male	30–65	Western Finland
G2-P3	Problem	Female	65 +	Western Finland
G3-P1	Problem	Male	30–65	Eastern Finland
G3-P2	Problem	Female	30–65	Southern Finland
G3-P3	Problem	Female	30–65	Western Finland
G3-P4	Problem	Female	30–65	Helsinki metropolitan area
G3-P5	Problem	Female	30–65	Eastern Finland
G4- P1	Problem	Female	30–65	Western Finland
G4-P2	Problem	Female	30–65	Helsinki metropolitan area
G4-P3	Problem	Female	30–65	Southern Finland
G4-P4	Problem	Female	18–29	Southern Finland
G6-P1	Recreational	Male	30–65	Western Finland
G6-P2	Recreational	Male	30–65	Western Finland
G7-P1	Problem	Male	30–65	Northern Finland
G7-P2	Problem	Male	30–65	Northern Finland
G7-P3	Problem	Male	30–65	Southern Finland
G7-P4	Problem	Male	65 +	Eastern Finland
G8-P1	Problem	Female	30–65	Eastern Finland
G8-P2	Problem	Female	30–65	Southern Finland
G8-P3	Problem	Female	65 +	Western Finland
I1	Problem	Female	30–65	Northwest Finland
I2	Problem	Male	30–65	Central Finland
I3	Problem	Male	18–29	Southeast Finland

### Analysis Methods

The analysis is based on a qualitative approach. We have used the deductive content analysis as method (Elo and Kyngäs, 2008, p. 111–112). We first read the data and defined the research question and focus. We then developed a categorisation matrix of the questions we asked the qualitative data (Table 3). Then we analysed the data by forming main categories and sub-categories. The main categories are (a) experiences of EGM closures; (b) experiences of EGM re-openings, (c) maintained habits; and (d) shifted habits. Groupings under these main categories are detailed in Table 3.

**TABLE 3:** Categorisation matrix.

Main categories	Questions	Sub-categories
Closures	What was the impact of EGM closures on gambling behaviour and habits? Why?	No impact, stopping gambling, relief, annoyance, positive health or financial outcomes
Re-openings	What happened when the EGMs were re-opened? Why?	No impact, recommencing gambling, gambling less than before, gambling more than before
Maintained habits	Will changed habits be maintained after the pandemic? Why?	Maintaining non-gambling on EGMs, maintaining new gambling habits (shifts)
Shifting habits	Did EGM gamblers shift to other gambling products during or after closures? Why?	No shifts, shifts to online products, shifts to other non-closed land-based products

The data were analysed using qualitative data analysis software Atlas.ti by two researchers. We analysed the answers to open-ended questions in the online questionnaire data as well as interviews by applying codes that correspond to the main themes of the categorisation matrix. Codes were attributed to full answers in the case of the questionnaire data and to full statements in the case of the interview data. While the study concerns experiences of EGM gambling, we also included answers by non-EGM gamblers when they were relevant to the matrix or described observations of the gambling of others (EGM gamblers). The results include excerpts from the interviews or survey responses to exemplify the analytical categories. Excerpts from interviews are referred to by the identifiers described in Table 2. Excerpts from the online survey are marked by the gender and age of respondents. All translations from Finnish or Swedish to English are by the authors.

In addition to the qualitative analysis, descriptive statistics are given of the prevalence of trends and responses in the data using percentages.

## Results

**FIGURE 1:**
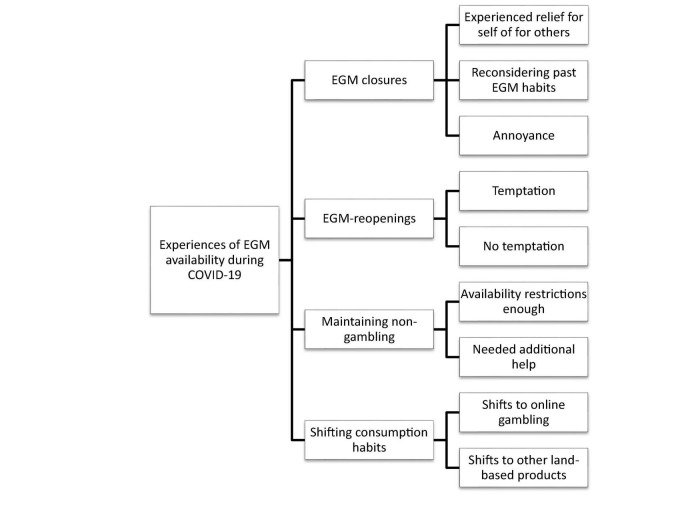
Analytical summary of the effects of experiences on EGM availability during COVID-19.

The results are presented in the following under four themes that correspond to the main categories in our deductive categorisation matrix (Table 3). The focus is on how the respondents experienced limited EGM gambling provision during the prolonged pandemic situation. The most discussed theme remained EGM closures. Re-openings were not discussed to a similar degree despite both questionnaires and interviews prompting respondents on both topics. This may be because during the time of data collection (early spring 2021) EGMs had again been closed in most Finnish regions to prevent the spread of the coronavirus. An overview of the main analytical categorisations of the analysis is presented in Figure 1.

### Experiences of Electronic Gambling Machine Closures

The online questionnaire asked respondents of changes in their monetary consumption on gambling during COVID-19. Twenty one percent of all respondents (including EGM and non-EGM gamblers) reported having spent less money on gambling during the pandemic, while 20 percent reported spending more. Fifty nine percent or respondents reported no change or did not want to respond. A closer look at the data shows that those who had reduced their gambling were or had been predominantly EGM players during or before the pandemic. Forty three percent of those who reported having played EGMs in the last 12 months had reduced their gambling consumption since the COVID-19 restrictions. The remainder had maintained their gambling consumption or increased gambling during the pandemic.

In the questionnaire and interview data, almost all participants expressed delight over EGM closures. Even respondents who did not personally gamble on EGMs noted that closures were positive for those who did. A small minority of respondents reported negative feelings about the closures because EGMs had for them been a non-problematic hobby, and “*it was nice to play a couple of euros now and then on [EGMs]*” (male, 35–40). One participant also felt that problems caused by EGM gambling were seen as a label that also prevents casual EGM gamblers from partaking in their hobby:

*The machines are closed so the obvious change has been that I can’t play. Some must be happy that they can save their money. Some lonely old people will likely be missing going out to see people and getting some excitement from gambling.* (female, over 74).

For the vast majority, however, the closure of EGMs was a relief. Closures had reduced time and money spent gambling and improved general welfare. One respondent explained how the shutting down EGMs had had *“a positive effect. The time I used to spend on those machines is now spent with the family”* (male, 35–49). Similarly, for many interviewed self-identified problem gamblers who had previously experienced EGM related harm, the shutdown of EGMs was experienced positively. Some no longer felt an urge to gamble, and many wished that the EGMs would be permanently closed or removed from non-casino settings, such as supermarkets:

*It was a relief. I would not mind if the EGMs would never be opened again. I often lost my coins in these games at the supermarket, and sometimes I withdrew money from my bank account to gamble. Usually, I went to the kiosk to play on EGMs because I did not dare to play too much at the supermarket. I was afraid to bump into my acquaintances and relatives. I knew that they did not go much to the kiosk and there I could play as much as I wanted to.* (G8-P2)

Similar stories were reported by participants in the online questionnaire. One respondent describes how *“peace has returned to my soul as I no longer gamble”* (male, 65–74). Even participants who did not personally gamble on EGMs had noticed the positive effects of closures. Respondents reported noticing that “*old people can now shop without being tempted to gamble”* (female, 65–74), or that “*there are less stressed, worried looking people around the machines”* (female, 50–64).

In the questionnaire data, respondents were asked whether non-casino EGMs should be reopened after the pandemic. Forty six percent of respondents wanted to maintain closures while 28% of respondents wanted EGMs to be reopened. The remainder had no opinion or suggested alternative options such as reducing the number of EGMs. One respondent explained his negative stance to re-openings by noting that he does not miss EGMs:

*I’ve stopped gambling on machines after the pandemic started, and I hope they will never be opened again. They have not been a problem for me, I gambled on EGMs maybe 7–8 times a year. But I really don’t miss them.* (male, 35–49).

Some participants had started to reflect on their gambling behaviour before COVID-19-related restrictions shut down EGMs. The felt ashamed of their compulsive behaviour, about lying to their family members, and of the many hours spent playing on EGMs. *Now I mostly feel more ashamed and anxious. My gambling behaviour has mostly been compulsive* (G7-P1), one recovering problem gambler explained in an interview.

Beyond reflection of individual behaviour, some respondents also felt that the societal acceptance of EGM gambling was shifting following restrictions in habitual availability. The wide availability of EGMs in non-casino settings has for long been a permanent fixture in Finnish supermarkets and petrol stations, and many had not reflected on their possible harms before COVID-19 changed this habitual pattern. “*Many have now started to understand how sick it is to have gambling machines in supermarkets and shopping centres”* (male, 25–34) one respondent explained. The same respondent continues later “*I have understood that I have been raised to be a gambler. Gambling had become a nasty habit*.” (male, 25–34). Some interviewees had come to similar realisations:

*When COVID started, we happened to be in Thailand. And there is nothing, actually [gambling] is prohibited in the whole country. It’s pretty cool. [In Finland], you go to the shop, and you look at people stuff coins in machines. It looks pretty awful. After I managed to stop, I have really started to pay attention to those machines when they were opened. There is not a single happy face playing those machines. There are no happy people.* (G2-P1)

### Experiences of Electronic Gambling Machine Re-Openings

While the closures of EGMs allowed for many EGM gamblers to stop gambling and reconsider their gambling, the re-opening of gambling opportunities appears to have served as a similar external shock that has allowed reconsidering one’s own gambling. Non-casino EGMs were opened in July 2020 but closed again in most regions again later in the autumn.

The online questionnaires asked how the re-opening of EGMs in July 2020 had impacted gambling. Of those who reported having gambled on EGMs during the past 12 months (*N* = 31), 55% indicated that their EGM gambling had returned to the same level as before the pandemic. Nineteen percent had played less than before while 26% of respondents reported having stopped gambling.

As our questionnaire only asked participant of past year gambling, the sample excluded those who had stopped EGM gambling when machines were first closed in March 2020. The qualitative analysis reveals that this group appears to have largely been able to maintain non-gambling despite describing re-openings as “*constant temptations”* (female, 50–64), or *“real tribulations”* (G4-P2). For others, the change of habit was less dramatic. Some described having “*already lost interest in EGM gambling anyway”* (female, 35–49), or having “*tried once, but then I realised it was pointless”* (male, 25–34).

Those who recommenced EGM gambling described “*immediately gambling a lot”* (male, 50–64). One self-identified recreational gambler in the interview data explained having travelled to another region where EGMs had been opened, was unable to resist them:

*I travelled to another region, and I noticed that EGMs were open. In my hometown they were closed. I immediately went to play. I was drunk when I was playing at a bar and that was it. I could say that when the EGMs will be opened again [in my region], it will only have negative consequences for me.* (G6-P2)

Re-openings have had even more severe consequences on those identifying as problem gamblers or as experiencing gambling harms. Some describe having played even more than before the pandemic. One participant recalls how he “*felt a horrible urge to constantly have an excuse to go out to the city to gamble”* (male, 35–49), while another describes having quickly hit rock bottom following excessive EGM gambling after re-openings:

*When the EGMs opened again last summer, I immediately went to play. It started with a coin, but then it got even worse than it was before. I know I will hit rock bottom even if it’s just one coin in the EGM. Playing is so addictive. In addition, EGMs are placed right at the entrance of the supermarket. Last summer I did not even have time to buy food when I had already played all my money. I borrowed a lot of money from everyone, and I sold my stuff a lot. All that I could get went down the EGMs because I thought that I could double my winnings.* (G4-P3)

### Maintained Habits

EGMs are designed to be addictive, and their visible placement in everyday environments may make it difficult to maintain non-gambling. In this section, we focus more closely on the experiences of those participants in the data who reported having gambled on EGMs previously but who had maintained non-gambling despite changing conditions. The levels of past-year EGM gambling differ across the respondents which is reflected in the qualitative results.

COVID-19 had allowed many to stop EGM gambling altogether. Most of these respondents had only gambled on EGMs sporadically or recreationally before the pandemic and had simply not picked up the habit since. “*I used to just play with a couple of coins. Now I don’t play at all”* (female, 65–74), one participant explained, while another one admitted that she had “*not even noticed that they were opened again”* (female, 35–49).

For others, and particularly for those who reported having previously experienced problems with their gambling, maintaining non-gambling had been somewhat more difficult, but still possible. For one participant, COVID-19-related restrictions had allowed them to *“finally break free from a gambling addiction that had lasted for 20 years”* (male, 35–49). Another reported having managed to “*keep away from machines since March 2020”* (male, 65–74). One participant explained that “*shutting down supermarket EGMs has reduced my urge to gamble significantly, and that is good. It’s the best thing that has come out of COVID-19”* (male, 25–34).

While changes in availability appear to have contributed to allow some to maintain the non-gambling habit, others explained that they had also needed additional help from gambling treatment and help associations or peer groups. Quitting gambling can be difficult, and changes in social environment and help from other recovering gamblers may be essential. One interviewee explained how COVID-19 as well as other help had made the seemingly impossible possible:

*I had been able to stop gambling [during the COVID-19 pandemic]. I would have never imagined it because I have gambled for 40 years. I am glad that I have been able to stop gambling. It is not so much online gambling, but I have always been tempted by EGMs. It was hard, and I got myself into financial trouble. I started to borrow money to gamble, and I still pay for it. I have not gambled for a year now. [*…*] There was this group in my city, I got help from there, and they had an exercise book that was good, really good.* (G2-P3)

The impacts of COVID-19-related restrictions were also confounded with other on-going changes in the Finnish gambling landscape that helped some maintain their non-gambling. For a small minority, negative public discussion, and increased criticism of the monopoly holder Veikkaus had impacted or ended their gambling. For others, mandatory identification for all EGM gambling, introduced in January 2021, played a role. Mandatory identification was seen as a way to stop gambling for two opposite reasons. Some respondents were in principle against being identified and refused to gamble if identification was required. *“Mandatory registration was the end of my EGM gambling. I will not accept anything mandatory”* (female, 65–74) one respondent highlighted, while another explained that *“I don’t want to identify with machines, it seems uncomfortable and too difficult”* (female, 35–49). Some respondents were also concerned over what Veikkaus would do with the data they collect. For others, identification was seen as a possible means to control one’s non-gambling in the future:

*What is positive is that I have informed Veikkaus that I am a gambling addict and Veikkaus has closed my account. I have also cut my Veikkaus card in half. My close ones know that I have a gambling problem. So, no one will lend me their Veikkaus card. As mine is cut in half, I cannot go and play on EGMs since identification is mandatory.* (G4-P3)

### Shifting Consumption Habits

In the online questionnaire data, those who described having shifted or having perceived shifted consumption habits in others away from EGMs following closures (*N* = 39) predominantly referred to gambling moving to online environments (82% or coded responses). Fifteen percent had shifted or perceived shifts in others toward still available land-based opportunities. The remainder merely referred to “other games” without specifying the channel or mode of participation. As already indicated in an earlier Finnish study conducted during the first months of the pandemic (Järvinen-Tassopoulos et al., 2020), increased free time and staying at home were highlighted as the main reasons for shifts to online gambling. Some gamblers have also substituted EGM gambling with other land-based gambling products that have remained available while EGMs were closed. The levels of gambling engagement again differ amongst these respondents, and these were considered in the qualitative analysis.

Shifted consumption toward other land-based products consisted mainly of scratch card or lottery gambling. Both types of gambling are available in the same supermarket and petrol station locations as EGMs and therefore constitute an easy substitute for convenience gambling. “*While the machines have been closed, I’ve put more money in the lottery”* (male, 50–64), one respondent explained. Another respondent had noticed that “*when the machines were shut down, there have been more people queuing [for lottery tickets and scratch cards] at the cashiers and at info desks”* (female, 65–74). However, none of the quotations describing shifts to land-based gambling expressed concern over possible problem behaviours related to this. Shifts to other land-bases products rather appeared to be connected to lower gambling involvement and lesser harms. Rather, shifts to slower games from EGMs were described as positive. “*I’ve been playing more horse betting games while being happy about EGM closures”* (male, 50–64), as explained by one survey respondent.

Those who had shifted their consumption toward online products had experienced more harms and reported to have also high gambling engagement. Some had not increased their consumption, but merely changed their mode of gambling. One gambler explained that as EGMs were closed and it was not possible to play while going to the supermarket, he had “*started playing online when he got back from the shops”* (male, 35–49). However, others explained that the constant availability of online gambling combined with addictive game designs were even more habit-forming than EGMs and shifts to online gambling had either created or exacerbated existing harms. Following the EGM closures, one respondent described that they had “*obviously reduced gambling on [EGMs], but then I moved online. It has taken even more of my money”* (female, 50–64). Online gambling has been connected to an increase in harms in comparison to land-based gambling also in previous research literature (Gainsbury et al., 2019; Raybould et al., 2021), and this was also the experience of many gamblers:

*My use of money went out of control in the autumn of 2020. I have overdrawn on two credit cards, and I have taken an instant loan [*…*]. During COVID-19, I started playing on foreign online casinos. I did not know before that I had a gambling problem, only in 2020 the situation became dramatically worse* (female, 35–49).

The questionnaire also inquired whether participants had gambled on Veikkaus or offshore provider products during the pandemic. Thirty percent of all respondents had also played on offshore gambling sites during the pandemic, while 16.5% had also played on the website of PAF. PAF is a gambling provider located on the autonomous Finnish Åland islands and it has been the main offshore gambling provider in Mainland Finland. In the last population study from 2019, its share of offshore gambling had nevertheless declined (Salonen et al., 2020). Many gamblers had also shifted their consumption to the monopoly holder Veikkaus’ online platform. Amongst those respondents who reported having gambled on EGMs in the last 12 months, 38% had gambled on offshore websites.

In the qualitative analysis, both offshore websites and Veikkaus websites were described as problematic due to their habit-forming nature. Some describe a “*constant urge to play on the Veikkaus website”* (female, 50–64):

*I’ve noticed that during teleworking I easily pick up my phone during my lunch break and go to the Veikkaus website. This never happened before* (female, 35–49).

The reasons for increases online gambling were discussed in the interview material. Participants explained that negative emotions, boredom, COVID-19-related anxiety, and lack of other leisure options had increased their urge to gamble:

*It has been so hard during the COVID-19 pandemic, because all the leisure activities have stopped, and all the places are closed. You cannot vent your bad feelings anywhere and then you open an online casino. You lose your money, and it frustrates you and you feel even worse. I gamble to escape my bad feelings. Gambling has always been my way of escaping. It is a bad one, but I have turned to it quite often.* (G7-P2)

*And then it is winter, and I do not like it at all, especially when it is gray and rainy. I work in the restaurant trade, and it has been hard for us [due to the COVID-19 pandemic]. All the stress I get from work and everything: I have noticed that it affects my gambling. I think about gambling a lot, and I gamble too much [online]. I gamble my salary.* (G8-P1)

A few gamblers also saw a positive side to online gambling, as it has allowed for better options to control and limit consumption:

*Luckily I realised to set a monthly spending limit. It might be that during my initial excitement [about online gambling], I might have spent tens of euros. It would have caused remorse and impacted my mental health. I get addicted easily, so I am very grateful for the opportunity to set limits in the app.* (female, 35–49).

## Discussion

This paper has focused on the experiences of EGM gambling (or non-gambling) 1 year into the COVID-19 pandemic. Results are aligned with those from previous studies: closures of EGM venues and non-casino EGMs have reduced total consumption and harms, and consumption has not been systematically substituted by other products (e.g., Lindner et al., 2020; Brodeur et al., 2021; Hodgins and Stevens, 2021). Some reductions have also been maintained after COVID-19 restrictions were lifted (Black et al., 2021). Observations from our sample have also been in line with previous Finnish findings (Järvinen-Tassopoulos et al., 2020; Taloustutkimus, 2020; Marionneau and Järvinen-Tassopoulos, 2021). Our qualitative analysis has also provided new information on EGM re-openings, and what kind of factors have contributed to either stopping gambling or shifting habitual consumption patterns in the Finnish context.

EGM shutdowns in Finland have changed the habitual landscape of gambling and forced (or allowed) gamblers to reconsider their relationship to these machines. Following the pragmatist understanding of the concept of habit, COVID-19-related closures have therefore constituted a new situation in which those who gamble have also adapted (cf. Kilpinen, 2012). For some of the participants in the current study, and notably those having previously gambled on EGMs, the closures have been an opportunity to break a long gambling habit. Changing conditions and situations caused by closures of habit-based EGM play have allowed our participants to reflect not only on their own gambling but also on the institutional context of gambling in Finland. Some had even grown increasingly critical of the wide availability of harmful gambling products in the Finnish society.

When EGMs were reopened, some participants reported having resumed their old behavioural patterns while others managed to maintain non-gambling. Some reported shifting their consumption to other gambling products, and particularly online gambling. Those who already gamble on fast, habit-forming EGMs appear to have preferred to substitute this gambling with other fast-paced, digital, and habit-forming gambling products, particularly online gambling. The result is aligned with an earlier German study (Georgiadou et al., 2021) showing that EGM gamblers had mainly substituted their habit with online EGMs. Previous research on the substitution effects across gambling products has also suggested that faster gambling games may be more easily substituted by other fast gambling games rather than by slow games such as lotteries (Marionneau and Nikkinen, 2017). Likewise, in our data, most EGM gamblers who reported having shifted consumption to new products were gambling other fast-paced online games and the online environment has exacerbated harms for some.

The results allow us to draw two policy-relevant conclusions. First, EGM provision is characteristically habit-forming. Habit-forming technologies are designed to maximise time on device (Schüll, 2012) while extensive exposure to gambling leads to increased consumption (Abbott et al., 2016; Markham et al., 2016). Habit-formation therefore becomes not only an in-game or in-app extension of time on device, but also a question of tempting consumers to access the service or game regularly (cf. Langvardt, 2019). An environmental cue encouraging habitual gambling may result in life-changing behavioural problems and harms. Changes in situational conditions appear to have been necessary for many to stop gambling on EGMs. Limiting exposure to harmful EGMs in everyday locations such as supermarkets also after COVID-19, as well as limiting habit-creating designs in EGM games is essential to reduce harms.

Second, shifting EGM consumption to online gambling can prove equally harmful. EGMs and online gambling are both fast paced products that have been connected to high levels of harms (Livingstone et al., 2019; Raybould et al., 2021). Effertz et al. (2018) have counted that a substitution of 10% of offline gambling with online gambling increases the probability of problem gambling by approximately 10%. While online gambling is not characterised by temptations to gamble in public or semi-public spaces, their convenience and accessibility, as well as the wide availability of platforms contribute to harms. Socially responsible gambling policies also beyond the pandemic need to consider and regulate habit-creating gambling availability also in online environments. This includes tacking harmful characteristics such as product design, 24 h availability, and additional incentives such as push notifications that encourage habit-based consumption.

The current study has been limited to studying the experiences of gamblers during COVID-19 in Finland. Finland has had a relatively lenient policy toward COVID-19 restrictions. While social distancing has been encouraged and many services have been closed, there have been no mandatory lockdown measures. Experiences of gamblers may be very different in other contexts with strict lockdowns or curfews. The study approach also has limitations. First, the qualitative nature of the study does not allow us to generalise the results to the entire Finnish population. Second, particularly geographical differences across participants from different Finnish regions could not be systematically studied with our sample size. Third, gamblers who participated in this study were self-identified recreational gamblers or problem gamblers (including those, who had succeeded to limit or stop their gambling) and we cannot draw conclusions on the diagnostic levels of problems or harm that they might be experiencing. Fourth, the study has focused on gambler experiences rather than actual behavioural changes in gambling patterns that could be better determined by for example utilising company sales or tracking data.

Further studies should also focus on the experiences of concerned significant others of gamblers as well as on the experiences of other products than EGMs. Comparative studies on the experiences of different gambler groups or across different societal contexts would be particularly needed. The current study has nevertheless been able to give a qualitative account of the different experiences related to EGM availability during the COVID-19 pandemic and how EGM policies during COVID-19 have contributed to turning habit-creating availability into habit-breaking.

## Data Availability Statement

The datasets presented in this article are not readily available because data analysis is still ongoing for other publications. Requests to access the datasets should be directed to VM, virve.marionneau@helsinki.fi.

## Ethics Statement

Ethical review and approval was not required for the study on human participants in accordance with the local legislation and institutional requirements. Participants over 18 years old consented to the study themselves. The only under 18-yearold participant participated anonymously in the web survey and was asked to click to confirm that they had permission from a guardian to participate.

## Author Contributions

VM and JJ-T were both in charge of data collection and the conception of this article, corrected the text, and gave their final approval. VM analysed the questionnaire data and wrote the first draft of the article. JJ-T analysed the interview data. Both authors contributed to the article and approved the submitted version.

## Conflict of Interest

The authors declare that the research was conducted in the absence of any commercial or financial relationships that could be construed as a potential conflict of interest.

## Publisher’s Note

All claims expressed in this article are solely those of the authors and do not necessarily represent those of their affiliated organizations, or those of the publisher, the editors and the reviewers. Any product that may be evaluated in this article, or claim that may be made by its manufacturer, is not guaranteed or endorsed by the publisher.
